# Power Spectral Analyses of Index Finger Skin Blood Perfusion in Carpal Tunnel Syndrome and Diabetic Polyneuropathy

**DOI:** 10.1155/2011/465910

**Published:** 2011-05-29

**Authors:** Han-Wei Huang, I-Ming Jou, Chien-Kuo Wang, Pei-Yin Chen, Wen-Chi Wang, Chou-Ching K. Lin

**Affiliations:** ^1^Department of Neurology, National Cheng Kung University Hospital, Tainan, Taiwan; ^2^Department of Orthopedics, National Cheng Kung University Hospital, Tainan, Taiwan; ^3^Department of Radiology, National Cheng Kung University Hospital, Tainan, Taiwan; ^4^Department of Internal Medicine, National Cheng Kung University Hospital, Tainan, Taiwan

## Abstract

The main purpose of this study was to investigate the applicability of frequency domain analysis on laser Doppler flowmetry (LDF) data recorded from the index fingers of patients with carpal tunnel syndrome (CTS) and diabetic polyneuropathy (DPN). Patients with numbness of the palm were recruited and grouped according to the results of electrophysiological examinations into 2×2 groups by the existence or nonexistence of CTS and/or DPN. Skin blood perfusion was recorded by LDF in both the neutral position and the maximally flexed position (the Phalen test). *S*-transformation was utilized to decompose the recorded data into frequency bands, and the relative band power and power dispersion were calculated. Analysis of variance was used to test the effects of DPN, CTS, and the Phalen test results. The results showed that (1) DPN decreased the absolute power and the relative power in some frequency bands in both positions and CTS increased the power dispersion of some frequency bands only during the Phalen test and (2) there was no difference in the LDF results between patients with positive or negative Phalen test results.

## 1. Introduction

Laser Doppler flowmetry (LDF) is an established technique to measure local cutaneous blood perfusion [1], which is simultaneously affected by cardiac pumping, respiration, control of the autonomic nervous system, and local metabolism. Because each factor has its own characteristic frequency, frequency domain analysis is an ideal technique to separate these factors and investigate their changes in different conditions [2]. When transformed to frequency domains, in the so-called power spectral analysis (PSD), LDF data can be decomposed into five components: 2–0.6 Hz (heart rate related, HR), 0.6–0.15 Hz (respiration rate related, RR), 0.15–0.06 Hz (myogenic, MUS), 0.06–0.02 Hz (neurogenic, NEU); and <0.02 Hz (metabolism related, MET) [3]. The frequency ranges and the physiological origins of these components are almost identical to those in heart rate variability (HRV) [4], except that the NEU and MET components are usually not separated in the latter. The differential effects on these components may be used to investigate the pathophysiological mechanisms of diseases. For example, while diabetes causes diabetic polyneuropathy (DPN) by reduced blood perfusion due to microvascular angiopathy [5], the compression of median nerves in carpal tunnel syndrome (CTS) may impair the autonomic control of local blood perfusion in the distal innervated area [6]. 

CTS, caused by the local compression of the median nerve in the carpal tunnel, is one of the most common neuropathies [7]. While the idiopathic form is more common in women of old age and those who are overweight, it is related to repetitive hand movements in younger populations [8]. The patients usually complain about numbness of the palm side of the hands, especially during repetitive manual tasks and during sleep. On the other hand, DPN, caused by diabetes mellitus (DM), is the most common form of polyneuropathy [9]. DPN is a sensory predominant neuropathy and the numbness is most prominent at the tips of toes and fingers. Even though the distribution and the characteristics of these two disease entities are different, some symptoms overlap and these two diseases may coexist. In fact, diabetes increases the prevalence of CTS. The prevalence of clinical CTS has been shown to be 2% in the reference population, 14% in diabetic subjects without DPN, and 30% in those with DPN [10]. Routine electrophysiological techniques, that is, nerve conduction velocity (NCV) studies, may not be able to detect the existence of CTS in the presence of DPN [11]. 

Existing data about frequency-domain analyses of LDF signals in DM and CTS patients are scarce. The main purposes of this study were firstly to investigate the applicability of PSD on LDF data recorded from the index finger pads of patients with CTS and DPN and secondly to evaluate whether PSD of LDF data can assist in the diagnosis of CTS in DPN patients. *S*-transformation [12, 13], a new time-frequency analysis tool, was adopted for calculating PSD.

## 2. Materials and Methods

### 2.1. Subjects and Experimental Procedures

The candidate subjects were recruited from the outpatient Clinics of Neurology, Orthopedics, and Internal Medicine of National Cheng Kung University Hospital (NCKUH). The inclusion criterion was the complaint of insidious onset of intermittent numbness of the palm. The exclusion criteria included stroke, structural abnormalities or trauma of the wrist, established cervical radiculopathy, liver and kidney dysfunction, autoimmune diseases, polyneuropathies, or other peripheral neuropathies with unknown etiology or known etiologies other than diabetes. The study protocol was approved by the NCKUH ethics committee on human subject study. Before an experiment began, the purpose, the potential hazards, and the experimental procedures were fully explained to the subject. All participants signed written informed consent forms. The subject's age and sex were recorded, and body weight and height were measured. 

The glycosylated hemoglobin (HbA1c) percentage of the serum was tested, and a value greater than 6.5% was used to confirm the existence of DM. Routine nerve conduction velocity studies, including F-wave latencies of the upper and lower limbs, were performed to help establish the diagnosis of carpal tunnel syndrome and polyneuropathy and exclude cervical radiculopathy. Short-distance comparative sensory nerve conduction studies were used to calculate the combined sensory index (CSI) for diagnosing milder CTS [14]. We adopted a grading system [15] to define the existence and severity of CTS. CTS with grades 2 to 6 could be established by routine NCV studies. For those subjects with normal routine NCV study results, CSI (>0.9) was used to establish grade 1 CTS. 

From the results of electrophysiological studies and blood tests, the candidate subjects were assigned to one of four groups by the existence of DPN and/or CTS, that is, EPSN: DPN(−)CTS(−), DPN: DPN(+)CTS(−), CTS: DPN(−)CTS(+), and DPN+CTS: DPN(+)CTS(+). The subjects that could not be fit into these groups were excluded.

### 2.2. Experimental Setup

**Figure 1:**
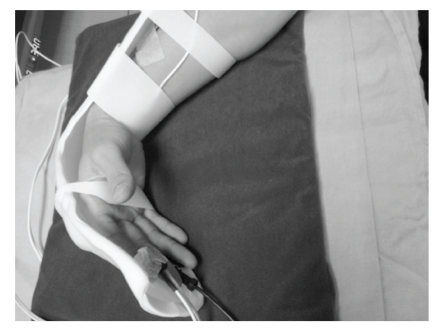
A hand of a subject with the custom-made apparatus to fix the wrist in the maximally flexed position.

The subjects were placed in a sitting position with the forearm and hand placed on a table. An electrogoniometer (TSD130B, Biopac System Inc., www.biopac.com) was fixed using tape across the dorsum of the wrist. The LDF probe (PF4001-2 Periflux Master, Perimed, www.perimed.se) was attached to the finger pad of the index finger. A thermometer (PF4005-2 PeriTemp Tissue Heater, Perimed, www.perimed.se) built into the LDF probe was used to keep the surface temperature of the finger pad at a constant value. An apparatus, custom made from a thermoplastic material commonly used for orthoses, was utilized to maintain the wrist in a maximally flexed position.  Figure 1 shows a hand of a subject with the apparatus. The hand and forearm were fastened to the apparatus with Velcro straps. The subject was instructed to push a switch with the other hand when the sensory symptoms appeared in the tested hand. Four signals, including the LDF signal, the switch state, the wrist angle, and the finger pad temperature, were digitized and recorded on a personal computer by a commercial analog-to-digital conversion system (MP100, Biopac System Inc., www.biopac.com) at a sampling rate of 200 Hz/channel and with a total duration of 6 minutes. Two sets of data were recorded: one with the wrist in the neutral position and the other with the wrist in the maximally flexed position. The latter position was to simulate the Phalen test maneuver [16].

### 2.3. Data Processing

The LDF signals *q*(*k*) were analyzed both in the time and the frequency domains. In the time domain, *q*(*k*) was first downsampled by 200 to *q*_*d*_(*k*), so that the sampling rate would become 1 Hz. The time average (Av) was calculated as the average over the whole recording period (360 seconds), that is,

(1) $$\text{Av}=\frac{1}{360}\overset{360}{\underset{k=1}{\sum }}q_{d}\left[k\right],$$

and the slope (Sp) or the trend was calculated as the slope estimated from the least-square linear regression,

(2) $$\text{Sp}={\left(\frac{1}{360}\overset{360}{\underset{k=1}{\sum }}k^{2}\right)}^{-1}\cdot \frac{1}{360}\overset{360}{\underset{k=1}{\sum }}k\cdot q_{d}\left[k\right].$$

For the frequency-domain analyses, *S*-transformation [12, 13] was adopted,

(3) $$\begin{array}{c} S\left[j,n\right]=\overset{N-1}{\underset{m=0}{\sum }}F\left[m+n\right]\cdot e^{-2\pi^{2}m^{2}/n^{2}}\cdot e^{i2\pi mj/N}\;n\neq 0,\\ S\left[j,0\right]=\frac{1}{N}\overset{N-1}{\underset{m=0}{\sum }}F\left[m\right]\;n=0, \end{array}$$

where *S* was the *S*-transformed result, *N* was the number of sampled frequencies, *F* was the Fourier transformation of the *N*-point time series *q*(*k*), *j*, and *n* indexed the time and frequency axes, respectively. *S*-transformation was an extension of the Fourier transformation for the time-frequency analyses. In this study, the time step was 5 ms and the frequency step was 0.0028 Hz. The advantage of *S*-transformation over short-time Fourier transformation is that, similar to wavelet decomposition, *S*-transformation has better simultaneous frequency and time resolution. The advantage over wavelet transformation is that interpretation is easier, because the independent variable is direct frequency without any approximation. *S* was a two-dimensional array of complex numbers. The mean power density over time was derived,

(4) $$A\left(j\right)=\frac{1}{tp}\overset{tp}{\underset{n=1}{\sum }}{\left|S\left[j,n\right]\right|}^{2},$$

where *t*_*p*_ = 72000. The power ratio (*Q*_*C*_) for the frequency band corresponding to HR, RR, MUS, NEU, and MET was calculated as

(5) $$Q_{C}=\frac{\sum_{k(fr(k)\;\in \;B_{C})}A\left(k\right)}{\sum_{k(fr(k)\in \;B_{T})}A\left(k\right)},$$

where *C* indexed one of the following bands: HR, RR, MUS, NEU, and MET, *B*_*C*_ was the corresponding frequency band (0.6–2 Hz, 0.15–0.6 Hz, 0.06–0.15 Hz and 0.0095–0.06 Hz, resp.), *B*_*T*_ was the total power over 0.0095–2 Hz and  *fr* was the sampled frequency in the *S*-transformation. The dispersion of power for the frequency band (*D*_*C*_) corresponding to HR, RR, MUS, and NEU was estimated as

(6) $$D_{C}=\frac{\sum_{k(fr(k)\;\in \;B_{P})}A\left(k\right)}{\sum_{k(fr(k)\;\in \;B_{C})}A\left(k\right)},$$

where *B*_*P*_ was the corresponding peak frequency band (0.6–1.6 Hz, 0.15–0.4 Hz, 0.07–0.1 Hz, and 0.02–0.05 Hz, resp.). All of the calculations were performed with Matlab commercial software (www.mathworks.com).

### 2.4. Statistical Analyses

Two-way analysis of variance (ANOVA) was used to test the effects of CTS and DPN on the differences of the time-domain parameters of LDF, respective band power ratios (*Q*_*C*_), and band power dispersions (*D*_*C*_) among the groups (ESPN, CTS, DPN, CTS+DPN). One-way ANOVA was used to test the effects of a positive Phalen sign on the differences of respective band power ratios among the groups. The significance level was set as *P* < .05. All statistical analyses were performed using StatView (SAS Institute Inc., Cary, NC, USA).

## 3. Results

In total, 72 subjects were included. The basic data are summarized in Table 1. There were more females in all groups, and the groups with DPN tended to be older. When the subjects with numbness before the test were excluded, the overall positive rate of the Phalen test was 20.9%.  Table 2 shows the distribution of subjects with CTS according to the severity of CTS. The subjects with DPN tended to have higher grades (*P* = .01). However, there was no significant difference of severity between the Phalen sign positive and negative groups (*P* = .29), and, by the chi-square test, there was no significant correlation between Phalen sign results and DPN (*P* = .18). 

**Figure 2:**
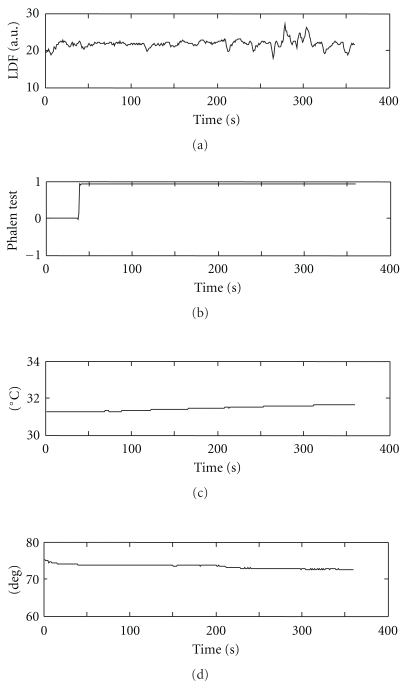
An example of original recordings. (a) Local blood perfusion from laser Doppler flowmetry, (b) a switch signal indicating the result of the Phalen test, (c) skin temperature of the index finger pad, and (d) wrist flexion/extension angle.

Figure 2 shows an example of recordings from the Phalen test. The first (uppermost) panel shows the LDF data, which fluctuated around 20 au (arbitrary unit) without an obvious trend. The second panel shows the subjective indicator of Phalen test results. In this case, the subject pushed the switch at around 40 seconds. The third panel shows the surface temperature of the index finger pad. The controlled target temperature was 32°C. The fourth panel shows the wrist flexion/extension angle. The angle decreased from 77° to 74° slowly with time, meaning that the wrist was less flexed. However, the angle change was small. 

**Figure 3:**
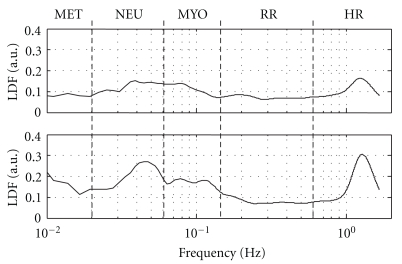
An example of transforming LDF results to frequency domain. Upper panel: neutral position and lower panel: Phalen maneuver. Abbreviations are explained in the text.

Figure 3 shows an example of frequency-domain analyses. The upper and lower panels show the results when the wrist was in a neutral and maximally flexed position, respectively. When the wrist was maximally flexed, the HR peak was consistently present and its amplitude was increased, the RR peak remained ambiguous, the amplitude of MUS base was increased, but no clear peak was present, and a peak appeared in the NEU band around 0.045 Hz. 

The results of analyzing LDF data with the wrist in the neutral position are summarized in Table 3. Two-way ANOVA with post hoc tests showed that the differences among the power ratios of the 4 groups were significant for *A*_HR_, *A*_RR_, and *A*_MUS_ for the factor DPN. The difference in Av was also significant for the factor DPN, while *A*_HR_ was larger in subjects with DPN, and *A*_RR_, *A*_MUS_, and Av were smaller. The results with the wrist in the maximally flexed position (Table 4) showed that the differences among the 4 groups were significant for *A*_MUS_, *A*_NEU_, and Av for the factor DPN. All *A*_MUS_, *A*_NEU_, and Av values were smaller in subjects with DPN. On the other hand, two-way ANOVA with post hoc tests (Tables 5 and 6) showed that the differences among the power dispersion of the 4 groups were not significant in any band for either factor in the neutral position and was significant for *A*_RR_ and *A*_NEU_ for the factor CTS in the maximally flexed position. *A*_RR_ and *A*_NEU_ were smaller, which meant the power was more dispersed, in the subjects with CTS. 

The effects of Phalen test results on LDF parameters were tested by one-way ANOVA with post hoc tests (Tables 7 and 8). Only Av was significantly affected by Phalen test results. Av was smaller when the Phalen test was negative compared to when it was positive. The results of statistical tests were similar for the wrist in the neutral and maximally flexed positions.

## 4. Discussion

Frequency-domain analyses can complement time-domain analyses in interpreting time array signals. Existing data about PSD of LDF signals in DM and CTS patients are scarce. The main reasons are probably that frequency domain analyses are less familiar and the tools are less commonly available to biological and medical fields. Traditionally, PSD was performed by Fourier transformation and extension to the time-frequency analyses, short-time Fourier transformation. Newer time-frequency analysis tools such as wavelet decomposition and *S*-transformation can not only perform PSD, but also localize specific changes of frequency powers in the time domain. As mentioned in Methods and Materials, the advantage of *S*-transformation over wavelet transformation is that the independent variable is direct frequency without any approximation. In this study, we averaged the band powers over time, so the results became equivalent to PSD. Still, the advantage of *S*-transformation is that the curve of PSD is smoother. 

Only one study about PSD of LDF signals in DM was found in the literature. The study, which measured LDF signals at the finger tip, reported that at the resting state, there was a significant difference in the absolute but not in the relative scalogram energy between the normal and the type I DM groups for all three frequency bands (MYO, NEU, and MET) [2]. These results are different from ours, which showed that the relative band powers of two bands (RR and MYO) were decreased and Av, which was equivalent to the absolute total power, was also decreased. Several differences in methodology might explain this difference. First, while the cited study recruited type I DM patients who were younger and without particular emphasis on the existence of neuropathy, we recruited type II DM patients with definite DPN and sensory symptoms. Second, the cited study recorded LDF at a lower sampling rate (3 Hz) for a longer time (1000 seconds), while we used a higher sampling rate (200 Hz) for a shorter time (360 seconds). A lower sampling rate saves storage space, facilitating a long recording time and increasing the resolution of lower frequencies, at the cost of losing information on higher frequencies and at a risk of aliasing. We used a higher sampling rate, so that all five known bands could be analyzed at the cost of a shorter recording time and a lower low-frequency resolution. Third, while the cited study used the total energy between 0.0095 Hz and 0.145 Hz for normalization, we used the total energy between 0.0095 Hz and 2 Hz for normalization. There are more existing data about frequency-domain analyses of HRV signals in DM patients. Stefanovska and Bračič [17] indicated that the human cardiovascular system is governed by five coupled oscillators. Thus, frequency-domain analyses of both heart rate variability and skin blood perfusion (LDF) reveal five similar frequency bands, though the controlling factors might be different. While skin blood perfusion is governed more by local factors, heart rate variability is influenced more by global factors. Though the affected frequency bands have been different, the common conclusion of studies on frequency domain analysis of HRV in DM patients has been that DM decreases the absolute and relative band powers in some frequency bands, especially HF and LF bands [18–24]. In fact, because the RR and MYO bands correspond, respectively, to the HF and LF bands of HRV, our results are compatible with the existing data from the literature about HRV in DM. In addition, because the statistical results were similar in both neutral and maximally flexed positions, our results imply that the blood perfusion was decreased by DPN but was not influenced by the increase of carpal tunnel pressure. The explanation is that main arteries to the fingers (radial and ulnar arteries) are exterior to the carpal tunnel and will not be directly compressed by the increase of carpal tunnel pressure. However, perfusion can be compromised due to the dysfunction of the autonomic fibers in the compressed median nerve. Our results also imply that for normal and DPN subjects, the increase of carpal tunnel pressure during the Phalen test was not high enough to compromise the function of autonomic fibers. In fact, there is a study indicating that nerve fibers in hyperglycemia are more resistant to ischemic conduction block during compression [25]. 

There is only one study about LDF in CTS patients, which is based on time-domain analyses. Schuind et al. [26] showed that, after carpal tunnel release, the arterial blood flow to the hand was increased. Our results showed that CTS did not affect the relative power ratio in any frequency band in either the neutral or the maximally flexed position. 

To the best of our knowledge, there are no documented studies about power dispersion in each frequency band. However, less power dispersion implies less variability of the frequency and less control action. Therefore, we developed this parameter to evaluate the control effort of the local cardiovascular system. In the maximally flexed wrist position, the tunnel pressure was expected to increase, leading to a decrease of blood supply to the median nerve and a compromise of its functions. However, the LDF results indicate that the absolute total power and the relative band power did not change in the maximally flexed position, while the relative power was more dispersed in the RR and NEU bands in the CTS group. The results imply that local perfusion did not decrease and maintained a similar oscillation pattern in both positions, although more control effort was needed to maintain the pattern in the maximally flexed position for the CTS patients. We think the possible explanations for the preserved control are that (1) the increase of carpal tunnel pressure during the Phalen test was not high enough to compromise the function of autonomic fibers or (2) the diameter of autonomic fibers is relatively small, and there were evidences [27, 28] that fibers of larger diameter were damaged first in CTS. Sp was very small for all groups, which also indicates that there was no particular trend during the recording course. 

Though some studies have indicated that the Phalen test is more reliable than other manual tests, the sensitivity (76%, ranging from 34 to 92%) and specificity (51%) are not satisfactory [29, 30]. Even in normal subjects, the Phalen test may be positive in 26% of subjects [31]. The main underlying mechanism of this test is believed to be due to the increased intra tunnel pressure during the wrist flexion, causing more compression of the median nerve. The Phalen test is inherently a qualitative test, because the compression pressure is not accurately defined. In addition, for those more severe patients with persistent numbness, the Phalen test is undefined, and for those patients with hypoesthesia, the results may be negative. One study reported that the Phalen test might be a better test for tenosynovitis than CTS [32]. All of these factors together may explain the low sensitivity and specificity of the Phalen test. 

The finding that Av, representing the total perfusion, was larger in patients with a positive Phalen sign (Tables 7 and 8) implies that the test might be more suitable for those with milder severity. As the disease exacerbates and Av decreases, the numbness becomes persistent. When the nerve functions are more compromised, hypoesthesia becomes predominant and the patients might not feel numbness. 

There are several limitations in interpreting the results of this study. First, the simple apparatus adopted in this study could not ensure that the wrist was maintained at the same angle throughout the experiment. However, because the drift was small and the tunnel pressure was a continuous function of wrist angle [33], the tunnel pressure was still markedly increased throughout the test. The results of Sp, which were very small for all groups, were also indicative that the small drift of the wrist angle might not have a significant effect on LDF recording. Second, the patients with DPN tended to be older, and there were more females in all groups except in the DPN group (Table 1). However, when these factors were set as confounding factors in the statistical analyses, the conclusions were similar. In addition, the review of Bircher et al. [34] indicated that age had no effect and sex had a minor or no effect on LDF recording of the skin. Third, LDF recording of a small area is characterized by a great temporal and spatial heterogeneity and, therefore, low reproducibility. The recently developed technique of laser Doppler perfusion imaging [35, 36] may partly ameliorate the problem, at the cost of longer recording time. It is also possible that the procedures developed in this paper can be extended to the data of perfusion imaging when the recording time and the size of recording area are properly chosen.

## 5. Conclusions

The results of this study showed that, in both the neutral and the maximally flexed wrist positions, the total perfusion and some components of power spectral analysis were significantly decreased in diabetic polyneuropathy but not in carpal tunnel syndrome, and, in contrast, some components of power spectral analysis were more dispersed in carpal tunnel syndrome in the maximally flexed position. In summary, while more research is needed, power spectral analysis of laser Doppler flowmetry data in conjunction with the Phalen test may be a new tool for studying carpal tunnel syndrome in diabetic polyneuropathy patients.

## Figures and Tables

**Table 1:** The basic data of the recruited subjects.

	EPSN	DPN	CTS	CTS + DPN
Number of subjects	11	12	25	24
Gender (M/F)	2/9	7/5	5/20	8/16
Age (years old) (SD)	52.9 ± 10.9	60.7 ± 9.1	52.8 ± 8.4	60.5 ± 9.1
Duration of DM (year)		12.2 ± 10.6		13.9 ± 9.5
HbA1c (%)	5.7 ± 0.7	7.9 ± 1.4	6.0 ± 0.5	8.4 ± 2.0
Body weight (kg)	67.9 ± 11.1	61.8 ± 11.6	62.1 ± 8.1	65.1 ± 12.5
Body height (m)	1.61 ± 0.07	1.62 ± 0.06	1.58 ± 0.06	1.58 ± 0.08
Phalen sign (−/+/#)	8/3/0	8/3/1	18/5/2	19/3/2

^#^: numbness appeared before the Phalen maneuver was performed.

EPSN: normal electrophysiological study.

DPN: diabetic polyneuropathy.

CTS: carpal tunnel syndrome.

**Table 2:** Severity of CTS in groups with CTS.

Grade	1	2	3	4	5
CTS	7	2	10	2	4
CTS+DPN	0	3	9	4	8

DPN: diabetic polyneuropathy.

CTS: carpal tunnel syndrome.

**Table 3:** Two-way ANOVA of power ratio with wrists in the neutral position.

	EPSN	DPN	CTS	DPN+CTS	*P*_CTS_^#1^	*P*_DPN_^#2^
*Q*_HR_	0.898 ± 0.073	0.990 ± 0.066	0.903 ± 0.082	0.951 ± 0.054	.453	.027*
*Q*_RR_	0.035 ± 0.031	0.021 ± 0.030	0.032 ± 0.029	0.019 ± 0.019	.675	.045*
*Q*_MYO_	0.031 ± 0.026	0.014 ± 0.013	0.027 ± 0.027	0.013 ± 0.019	.637	.009*
*Q*_NEU_	0.027 ± 0.021	0.022 ± 0.028	0.030 ± 0.035	0.014 ± 0.016	.690	.126
*Q*_MET_	0.013 ± 0.012	0.017 ± 0.028	0.013 ± 0.015	0.006 ± 0.006	.148	.636

Sp	0.010 ± 0.077	0.008 ± 0.019	0.018 ± 0.046	0.001 ± 0.037	.996	.694
Av	49.04 ± 27.50	31.30 ± 20.02	43.18 ± 34.22	25.85 ± 15.01	.578	.020*

*: significant difference (*P* < .05) by two-way ANOVA.

^#1^: *P* value for the influence of the factor CTS.

^#2^: *P* value for the influence of the factor DPN.

**Table 4:** Two-way ANOVA of power ratio with wrists in the maximally flexed position.

	EPSN	DPN	CTS	DPN + CTS	*P*_CTS_	*P*_DPN_
*Q*_HR_	0.881 ± 0.060	0.927 ± 0.059	0.900 ± 0.082	0.927 ± 0.083	.629	.058
*Q*_RR_	0.042 ± 0.028	0.028 ± 0.034	0.031 ± 0.023	0.028 ± 0.040	.528	.300
*Q*_MYO_	0.034 ± 0.020	0.018 ± 0.016	0.030 ± 0.026	0.018 ± 0.020	.789	.013*
*Q*_NEU_	0.036 ± 0.022	0.019 ± 0.015	0.031 ± 0.035	0.019 ± 0.024	.721	.036*
*Q*_MET_	0.013 ± 0.009	0.014 ± 0.027	0.019 ± 0.020	0.030 ± 0.065	.301	.539

Sp	0.006 ± 0.026	−0.003 ± 0.027	−0.008 ± 0.045	0.009 ± 0.048	.939	.694
Av	49.59 ± 24.61	31.25 ± 22.25	43.96 ± 31.59	31.65 ± 15.75	.676	.016*

*: significant difference (*P* < .05) by two-way ANOVA.

**Table 5:** Two-way ANOVA of power dispersion with wrists in the neutral position.

	EPSN	DPN	CTS	DPN + CTS	*P*_CTS_	*P*_DPN_
*D*_HR_	0.956 ± 0.048	0.958 ± 0.034	0.957 ± 0.029	0.921 ± 0.097	.260	.287
*D*_RR_	0.698 ± 0.133	0.650 ± 0.102	0.654 ± 0.111	0.618 ± 0.115	.192	.151
*D*_MYO_	0.458 ± 0.034	0.437 ± 0.046	0.458 ± 0.071	0.442 ± 0.069	.875	.260
*D*_NEU_	0.369 ± 0.129	0.434 ± 0.147	0.405 ± 0.132	0.388 ± 0.155	.881	.511

**Table 6:** Two-way ANOVA of power dispersion with wrists in the maximally flexed position.

	EPSN	DPN	CTS	DPN + CTS	*P*_CTS_	*P*_DPN_
*D*_HR_	0.949 ± 0.057	0.954 ± 0.036	0.958 ± 0.031	0.919 ± 0.087	.400	.279
*D*_RR_	0.746 ± 0.144	0.715 ± 0.078	0.645 ± 0.124	0.637 ± 0.112	.004*	.517
*D*_MYO_	0.427 ± 0.055	0.453 ± 0.084	0.461 ± 0.064	0.470 ± 0.065	.133	.319
*D*_NEU_	0.529 ± 0.093	0.441 ± 0.192	0.421 ± 0.130	0.408 ± 0.126	.044*	.145

*: significant difference (*P* < .05) by two-way ANOVA.

**Table 7:** Effects of the Phalen test result on power ratios with wrists in the neutral position.

	Negative	Positive	Persistent	*P*
*A*_HR_	0.848 ± 0.061	0.834 ± 0.076	0.901 ± 0.025	.126
*A*_RR_	0.614 ± 0.069	0.600 ± 0.053	0.577 ± 0.084	.424
*A*_MYO_	0.359 ± 0.034	0.367 ± 0.025	0.373 ± 0.024	.450
*A*_NEU_	0.337 ± 0.046	0.348 ± 0.026	0.320 ± 0.041	.432

Sp	0.107 ± 0.446	0.130 ± 0.257	0.125 ± 0.159	.977
Av	31.49 ± 25.65	46.97 ± 27.93	42.70 ± 24.42	.010*

*: significant difference (*P* < .05) by one-way ANOVA.

**Table 8:** Effects of the Phalen test result on power ratios with wrists in the maximally flexed position.

	Negative	Positive	Persistent	*P*
*A*_HR_	0.837 ± 0.064	0.813 ± 0.068	0.878 ± 0.040	.134
*A*_RR_	0.627 ± 0.068	0.618 ± 0.057	0.598 ± 0.088	.622
*A*_MYO_	0.369 ± 0.034	0.364 ± 0.027	0.371 ± 0.020	.819
*A*_NEU_	0.336 ± 0.057	0.334 ± 0.052	0.360 ± 0.028	.634

Sp	0.033 ± 0.374	−0.111 ± 0.310	−0.042 ± 0.268	.363
Av	33.30 ± 19.75	52.49 ± 33.39	48.08 ± 30.81	.018*

*: significant difference (*P* < .05) by one-way ANOVA.
